# Efficacy and safety of Mudan granules for painful diabetic peripheral neuropathy: A protocol for a double-blind randomized controlled trial

**DOI:** 10.1097/MD.0000000000028896

**Published:** 2022-03-11

**Authors:** Aixia Zhang, Qian Wang, Min Liu, Mengxia Tan, Xiaodan Zhang, Raoping Wu

**Affiliations:** a Department of Physiology, Basic Medical College, Jiangxi Health Vocational College (Nanchang Medical College), Nanchang, Jiangxi Province, China; b Academic Affairs Department, Jiangxi Health Vocational College (Nanchang Medical College), Nanchang, Jiangxi Province, China; c Department of Pharmacology, Basic Medical College, Jiangxi Health Vocational College (Nanchang Medical College), Nanchang, Jiangxi Province, China.

**Keywords:** Mudan granules, painful diabetic peripheral neuropathy, protocol, randomized controlled trial

## Abstract

**Background:**

As one of the most challenging complications in the management of diabetes mellitus, painful diabetic peripheral neuropathy (PDPN) is accompanied by various clinical manifestations, including numbness, burning, coldness, and other sensory abnormalities in the extremities. Meanwhile, PDPN seriously affects the life quality of patients and causes great pain. Western medicine mostly provides symptomatic treatments, such as antioxidants, aldose reductase inhibitors, nerve nutrition, microcirculation improvement, and analgesic drugs on the basis of blood sugar control. Although certain efficacy has been achieved, the problem has not been solved at root. Mudan granules have some advantages in the treatment of PDPN, but there is insufficient high-quality clinical studies to verify this. Therefore, the purpose of this study was to evaluate the efficacy and safety of Mudan granules in treating PDPN.

**Methods:**

A randomized, double-blind, placebo, and parallel-controlled trial design was used to study the efficacy and safety of Mudan granules in the treatment of PDPN. In this study, 93 patients with painful diabetic neuropathy were recruited and randomly divided into a treatment group and a placebo group based on 1:1. The treatment group was given Mudan granules and the control group accepted placebo treatment, and the basic treatment was performed according to the recommended guidelines. During the treatment period, the patients’ visual analog scores, clinical efficacy, Medical Outcomes Study 36-item Short-Form Health Survey (SF-36) scores, nerve conduction velocity, and drug-induced adverse reactions were observed at baseline after 8 and 10 weeks.

**Discussion:**

This study will evaluate the efficacy and safety of Mudan granules in treating PDPN. The experimental results will provide evidence support to treat PDPN with Mudan granules.

**Trial registration:**

DOI 10.17605/OSF.IO/5CE32.

## 1 Introduction

Painful diabetic peripheral neuropathy (PDPN) is one of the common chronic complications of diabetes. Symptomatic DPN occurs in 50% of patients with diabetes, and about 15% of patients have symptoms that are quite severe, thus requiring treatment.^[1–3]^ The incidence of PDPN ranges from 10% to 16% depending on the diagnostic criteria.^[1]^ The clinical manifestations include burning, electric shock, acupuncture or blunt pain, more at night, fatigue or excitement when aggravating, often accompanied by hypersensitivity.^[4]^ PDPN patients usually suffer from depression and sleep disorders that seriously affect their daily life and quality of life.^[5,6]^

There is no clinically definitive efficacious treatment, so a comprehensive treatment must be taken. According to western medicine, the treatment of PDPN is based on blood sugar control, while improving microcirculation, aldose reductase inhibitors, antioxidants, ion channel inhibitors, nerve nutrition, tricyclic antidepressants, opioid analgesics, and antiepileptic drugs.^[7]^ However, even with the above mentioned drugs, the pain in PDPN patients still cannot be relieved. Currently, the pain caused by diabetic peripheral neuropathy is one of the world's most difficult problems in the treatment of pain, and there exist limitations in solely relying on modern medical treatments. Therefore, the advantages of traditional Chinese medicine (TCM) can be fully utilized, combined with Western medicine.

In recent years, some studies have revealed that TCM has advantages in treating PDPN with fewer adverse reactions.^[8]^ Mudan granules are composed of radix paeoniae root, *Salvia miltiorrhiza*, *Ligusticum chuanxiong*, red flower, rhizoma corydalis, Sappanwood, *Astragalus membranacea*, *Panax notoginseng*, and caulis spatholobus.^[9]^ Modern studies have proved that Sappanwood, Rhizoma corydophila, caulis spatholobi, *P notoginseng*, and *S miltiorrhiza* all have antioxidant, anti-inflammatory, and vascular protection effects.^[10–14]^ Rhizoma corydalis and *L chuanxiong* have the effects of promoting Qi and activating blood, and removing stasis and relieving pain; caulis spatholobus, Sappanwood, and miltiorrhiza can enhance blood circulation and stasis; *P notoginseng* can help blood circulation and pain; Astragalus is intended to enrich Qi and promote blood circulation, nourish blood, and achieve the effects of removing blood stasis. The combined use of all drugs invigorate Qi and promot blood circulation, and clear collaterals and relieve pain, which suits PDPN pathogenesis of Qi deficiency and blood stasis.

The treatment of PDPN by Mudan granules is clinically effective. It has been proved that Mudan has protective effect on the structure and function of nerve tissue in animal experiments,^[15–17]^ because these conclusions are based on animal experimental studies and control studies of low methodological quality. At present, there is no rigorous randomized controlled trial to investigate the efficacy and safety of Mudan granules in the treatment of PDPN. Therefore, we will evaluate the efficacy and safety of Mudan granules in treatmenting PDPN through this randomized controlled trial.

## 2 Materials and methods

### 2.1 Study design

**Figure 1:**
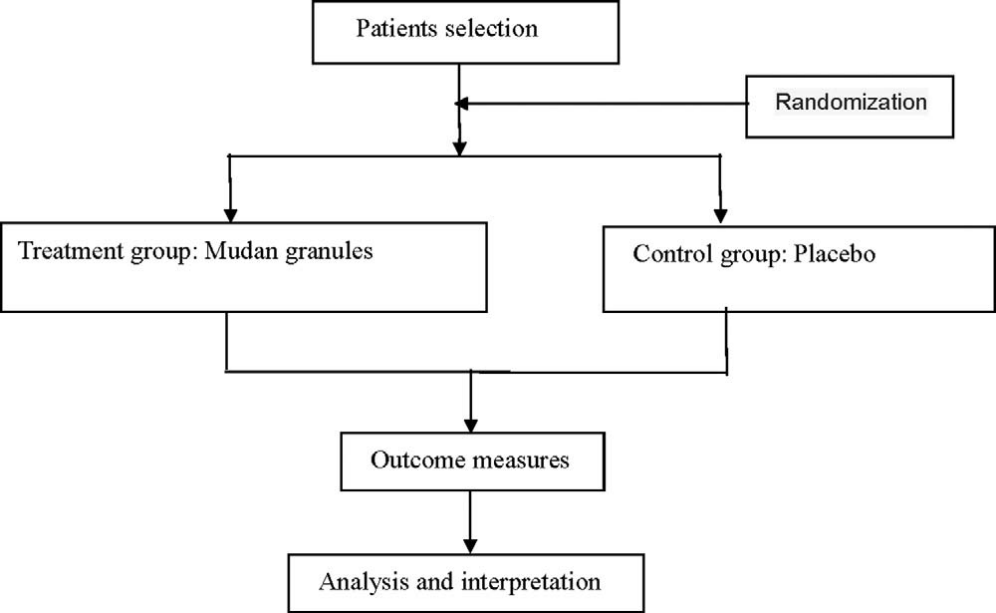
Flow diagram of the trial.

This is a randomized, double-blind, and placebo-controlled design. A total of 93 patients with painful diabetic neuropathy recruited in this study will be randomized at the ratio of 1:1 to receive treatment and placebo groups for 8 weeks, and the evaluators will assess and analyze the outcomes at 3 time points (pre-treatment, 8 and 10 weeks). The Jiangxi health vocational college will be responsible for data management and statistics. The flow chart of the trial is shown in Fig. 1.

### 2.2 Ethics and registration

This research protocol were conducted in accordance with the Declaration of Helsinki and Ethical Guidelines for Clinical Research and approved by our Clinical Research Ethics Committee. The protocol conforms to the Standard Protocol Items: Recommendations for Interventional Trials (SPIRIT) 2013 Statement,^[18]^ and the results were reported on the basis of the Consolidated Standards of Reporting Trials (CONSORT) Statement extension for trials.^[19]^ This experiment has been registered in the open science framework (OSF) (registration number: DOI 10.17605/OSF.IO/5CE32). All patients and their families signed a written informed consent form before the study, and they could discontinue or withdraw from the study at any time during the study.

### 2.3 Patients

#### 2.3.1 Participant recruitment

The recruitment information will be published on the hospital website, WeChat public website, Facebook, Weibo, and other official platforms to recruit potential patients. The patients who meet the inclusion criteria will have their personal data recorded in detail by the study staff. Moreover, the patients will be replaced by a number and managed by a dedicated person. All information will be stored in a database that can only be accessed by the staff responsible for randomization and blinding.

#### 2.3.2 Inclusion criteria

(1) Patients diagnosed with type 2 diabetes according to the criteria of WHO in 1999, aged from 40 to 70;
(2) Patients presented with obvious spontaneous neuralgia and numbness and other neuropathic symptoms, which were obvious in both lower limbs, and the pain was like burning knife to cut worms and dragging and needling, often accompanied by aggravation at night and/or sleep disturbance. Most importantly, the symptoms lasted for >6 months;
(3) Electromyography showed a decrease in motor sensory nerve conduction velocity (MNCV <45 m s^−1^, SNCY <45m s^−1^);
(4) Patients with pain visual analogue score (VAS) >4, without analgesic medication in the last 1 week;
(5) Michigan neuropathy screening instrument (MNSI) >2 point.
(6) Fasting blood glucose <8 mmol/L; 2 hours postprandial blood glucose <15 mmol/L, glycosylated hemoglobin <8.5%.

#### 2.3.3 Exclusion criteria

(1) Combined with other diseases, injuries, or drug taking history that may lead to peripheral neuropathy, such as pernicious anemia, hypothyroidism, and lower extremity vascular diseases (disappearance or weakening of dorsal foot artery fluctuation, varicose veins, etc); history of lower extremity trauma or surgery; drug use history such as vincristine and phenytoin sodium; history of heavy metal poisoning.
(2) Presence of abuse of alcohol or drugs, or malnutrition in one recent year.
(3) Previous history of cervical and lumbar spondylosis and radiculopathy.
(4) Pedal edema.
(5) Severe hemorrhagic disease or bleeding tendency.
(6) Intolerable to surgery or drugs due to vital organ dysfunction.
(7) Poor compliance.
(8) Rheumatism and rheumatoid diseases.
(9) Participating in other clinical trials.

## 3 Randomization and blinding

The random sequence numbering will be obtained by an independent researcher via www.randomization.com. Researchers will place the generated list of random numbers in sequentially numbered opaque sealed envelopes. The consecutive participants will be randomly assigned to the treatment or placebo group at the ratio of 1:1 based on the information obtained from the envelope. Each patient who met the inclusion criteria was randomly assigned a specific number, which was consistent with the specific number of the drug formulation used. Patients in both groups were given herbal granules for treatment. The study drugs (Mudan granules and placebo) were supplied by Aoda Pharmaceutical Co., Ltd. (Liaoning, China) in the corresponding granules, which was consistent in packaging, dosage, taste, and odor. In this regard, all patients and physicians do not know the group assignment to avoid bias.

## 4 Interventions and comparison

Both groups will receive routine diabetes care and treatment during the whole 8-week study. In 2011, the American Academy of Neurology published “Guidelines for the Treatment of Painful Diabetic Neuropathy” based on evidence from evidence-based medicine, which suggested that pregabalin treatment for painful diabetic neuropathy has Class A evidence. A systematic evaluation revealed that pregabalin treatment is a safe and effective treatment option for painful diabetic neuropathy in terms of suppressing pain, improving sleep therapy, and enhancing the body's tolerance.^[20–22]^ Based on all conventional treatments, including diet control, moderate exercise, stable blood glucose control, blood pressure and lipid control, and symptomatic treatment of comorbidities in both groups, except for the herbal granules, in the treatment period, the treatment regimen was divided into treatment and placebo groups according to randomization. All subjects in both groups were prohibited from using other herbal or proprietary Chinese medicines during the trial. To improve and monitor adherence, those who completed all treatments and assessments were financially compensated. In addition, each treatment form and assessment form will be completed and signed by the patients and the investigators.

### 4.1 Mudan granule group

We plan to use TCM granules at the beginning of treatment. All subjects will receive Mudan granules 3 times a day, 30 minutes after meals, for 8 weeks.

### 4.2 Placebo group

The placebo will be provided by AODA Pharmaceutical Co., Ltd. (Liaoning, China), and the placebo group will use grain-based excipients without corresponding pharmacological effects, and the dosage precautions will be the same as those for the treatment group.

## 5 Sample size

By reviewing the relevant literature,^[23]^ the independent statistician obtained the endpoint mean pain score (MPS). The mean value of the effects of the placebo group was 6.58; the standard deviation of the sample of the placebo group was 1.58; the mean value of the effects of the treatment group was 5.6; the standard deviation of the sample of the treatment group was 1.58. It is logged on the sample size calculation website (https://www.biostats.cn/statx/compute.html), and the relevant parameters were set. The 1-β test efficacy was 0.8 at *α* = 0.05 test level, the ratio of the sample size of the test group to the control group was 1:1, and 42 cases were obtained for the placebo group and the treatment group, respectively. Considering a shedding rate of no >10%, it was designed to include cases, and at least 93 patients should be included in this final clinical trial.

## 6 Observation indexes

(1) Clinical efficacy was formulated with reference to practical endocrinology and other relevant references. Apparent effects: Disappearance or significant improvement of clinical symptoms such as pain and numbness in the extremities, restoration, or significant improvement of knee and tendon reflexes, increase of nerve conduction velocity >5 m/s compared with that before treatment. Effects: The reduction of clinical symptoms such as pain and numbness in the extremities, partial restoration of knee and tendon reflexes, and the increase of nerve conduction velocity ≤5 m/s than before. In-effects: No improvement in clinical symptoms such as pain and numbness in the limbs, no improvement in knee and tendon reflexes, and no change in nerve conduction velocity. Total effective rate = (number of effective cases + number of effective cases) ÷ total number of cases × 100%.

(2) Nerve conduction velocity: NDI-092 electromyography was used for neurophysiological testing. Patients were placed in a supine position and the motor conduction velocity (MNCV) of the common peroneal nerve, median nerve, ulnar nerve and tibial nerve, and the sensory conduction velocity (SNCV) of the superficial peroneal nerve and median nerve were measured in a quiet state.

## 7 Safety

### 7.1 Primary safety parameter

The primary safety parameter was nerve conduction (NC) and assessed at 3 time points during the study period-baseline, endpoint, and follow-up. The mean NC measurements were compared at baseline and endpoint to determine if there were any changes in NC of nerve fibers during the study period. NC testing was carried out during follow-up to determine if any NC neurological deficits observed at endpoint were reversible 2 weeks after the end of drug treatment. All patients were required to complete the follow-up NC testing, regardless of whether they participated in this study in its entirety.

### 7.2 Secondary safety parameter

Patients’ routine blood, electrocardiogram, and liver and kidney function tests will be checked at baseline, endpoint, and follow-up of the trial, respectively, aiming to assess the safety of the treatment. All information on adverse events will be recorded at all times, including symptoms of adverse reactions, duration, severity of symptoms, management measures, etiology, and patient prognosis and regression. Investigators record, organize, analyze, and summarize in details.

## 8 Data collection and management

The study staff will collect basic subject information, observational indicators, laboratory indicators, and adverse events. Data collators will fill in the above data in the case report form and study medical record. The clinical trial data are traceable and require original information to be stored in clinical charts and databases, with a uniform collective storage method for all case information. All subjects will be replaced by number and no personal information shall be displayed. The clinical trial quality management specifications are strictly enforced throughout the clinical trial process.

### 8.1 Statistical analysis

The data statistician of Jiangxi health vocational college of Health Professions will apply SPSS 26.0 (IBM Corp., Armonk, NY, USA) software for data processing. Furthermore, the data statisticians will use descriptive statistics to summarize the baseline situation of the 2 groups. According to the type of data information, we will use the mean and standard deviation to describe continuous variables, dichotomous variables will use the chi-squared test, and rank data will be expressed by non-parametric test. *P* < .05 has statistical significance of differences.

## 9 Discussion

The pathogenesis of PDPN is still unclear, and scholars hold the view that it is multifactorial.^[24]^ Lower limb symptoms are more common than the upper limb, the disease is stabbing pain, burning pain, and drilling pain. Sometimes, severe pain such as amputation pain was aggravated day and night, which could cause foot ulcers, neuroarthritis, and life-threatening.^[25,26]^ Because pain involves central and peripheral mechanisms, there is no specific clinical treatment. Therefore, drugs are mainly used to relieve pain and control the disease.

From the perspective of TCM, the pathogenesis of PDPN is Qi deficiency and blood stasis.^[27]^ The disease is caused by the deficiency of Qi and Yin, obstruction of arteries and veins, and loss of nourishment of tendons. Thus resulting in impeded blood flow of the body, obstruction of arteries and veins, and pain, with clinical numbness of limbs and pain. The principle of treating diseases is to improve Qi and remove blood stasis, relieve pain, and promote blood circulation and collaterals.^[17]^

Mudan granule has the effects of invigorating Qi and relieving pain, promoting blood circulation and removing stasis, and clearing collaterals and relieving pain.^[28,29]^*L chuanxiong* and Rhizoma corylae have the functions of activating blood circulation and removing stasis, Qi and relieving pain. The compatibility of *S miltiorrhiza*, Caulis spatholobi, and Sappanwood can enhance blood circulation and remove stasis. At the same time, with the *P notoginseng* for activating blood and relieving pain and the astragalus for nourishing qi and nourishing blood, it has the effects of removing blood stasis and creating new, and can effectively improve the pain symptoms of patients.

The treatment of PDPN by Mudan granules is not only clinically effective, but also proved to have protective effect on the structure and function of nerve tissue in animal experiments.^[15–17]^ As these conclusions are based on animal experimental studies and controlled studies with low methodological quality, there were no rigorous clinical studies on evaluating the efficacy and safety of Mudan granules in the treatment of PDPN. Therefore, this study intends to explore the efficacy and safety of Mudan granules in the treatment of PDPN through a standard randomized controlled trial.

However, there are still some problems. The sample size of clinical studies is too small to provide sufficient evidence-based medical evidence. It is expected to carry out large-sample, multicenter, and prospective studies in the future. The depth of experimental research on its mechanism of action is not enough, and there still lack researches on the effective components of TCM and the interaction between components in the formula. Therefore, the mechanism of Mudan granule in the treatment of PDPN can be deeply discussed from multiple perspectives and links such as signal pathways.

## Author contributions

**Conceptualization:** Aixia Zhang.

**Data curation:** Qian Wang.

**Formal analysis:** Qian Wang.

**Funding acquisition:** Aixia Zhang.

**Investigation:** Min Liu.

**Methodology:** Min Liu, Mengxia Tan.

**Project administration:** Aixia Zhang.

**Resources:** Min Liu, Mengxia Tan.

**Software:** Mengxia Tan.

**Supervision:** Aixia Zhang.

**Validation:** Xiaodan Zhang, Raoping Wu.

**Visualization:** Xiaodan Zhang, Raoping Wu.

**Writing – original draft:** Aixia Zhang, Qian Wang.

**Writing – review & editing:** Aixia Zhang, Qian Wang.
